# Biology and Criminology: Data Practices and the Creation of Anatomic and Genomic Body ‘Types’

**DOI:** 10.1007/s10612-023-09732-6

**Published:** 2023-10-09

**Authors:** Mareile Kaufmann, Maja Vestad

**Affiliations:** Department of Criminology and Sociology of Law, University of Oslo, Kristian Augusts Gate 17, 0164 Oslo, Norway

## Abstract

The use of biometrics for the creation of visual ‘body types’ needs continued criminological engagement. This article discusses Lombroso’s practice of typing ‘born criminals’ vis-à-vis genomic phenotyping used to identify potential suspects. Both are prevalent examples of scientizing police and legal work. While Lombroso draws on anatomy to explain causes of criminal behavior, phenotyping is based on genomic and physiognomic correlation to help identify suspects. Despite these differences, both forms of visualizing bodies, we argue, are also a practice of *marking*. Especially in the context of crime and crime control, marking is a sensitive and political practice. Since typing is embedded in criminology, our analysis is also a critical engagement with criminology itself.

## Introduction

In 2017, the Orlando Police Department made an arrest in the case of 25-year-old Christine Franke, who was murdered in 2001. DNA played a major role here. Among other things, the department used a technique called phenotyping and created a facial image based on DNA found at the crime scene. It revealed “a black man with brown eyes, black hair and no freckles” (Lotan 2018). Though it was a different DNA-based technique that eventually identified a suspect, the image of the body type ‘black man’ circulated in the domain of crime governance. The imaging of body types, we argue, is a practice of crime control that keeps surfacing in different forms. These practices need criminological assessments.

Indeed, crime and control cannot be divorced from visual representation (Ferrel and Van De Voorde 2010). Visuals create, summarize and convey knowledge about crime. They show up as infographics and pictures in media (Wheeldon 2021), as hotspots on maps for predictive policing (Kaufmann et al. 2019), or as flags in data systems for national security (Amoore 2011). Visuals are pedagogical tools; they inspire new “reading practices” (Horn 2003:2) and render knowledge accessible in ways that are different from narrative presentations. Many, but not all, visuals are developed for professionals to support efficient decision-making in complex environments. This also means that such visuals have a tendency to simplify, to reduce complicated explanations to an image, a pattern or a type that can easily be grasped.

This article focuses on the visual language of the body. A biometric image for identification, so the logic goes, does not require the text or verbatim otherwise needed to identify a person. Instead of having to analyze a narrative that may be right or wrong, the body conveys the message that ‘it does not lie’ (Franko Aas 2006). That there seems to be something inherently ‘telling’ about the body is reflected in practices of crime control that identify corporeal patterns and translate them into profiles and types. Profiling practices are particularly sensitive in the contexts of law enforcement and forensics, because they ascribe a legal or an investigative status to entire groups. They *mark* body types. This can become a practical problem, for example when a profile takes investigations into the wrong direction. More important, biometric profiles live a social life that is no longer under the control of those who develop them. Profiles can become problems of discrimination (Magnet 2011). They affect identities and self-conceptions (Benjamin 2019). These politics of visualization are what we discuss in this article.

Visual profiles of bodies never emerge out of nowhere. They are a socio-technical practice. They are embedded in the history of explaining crime and the scientization of police and legal work. This history is entangled with the history of criminology per se—something that the discipline cannot cease to address. For that reason, we compare two particular forms of visual profiling: Lombroso’s biometric profiles, and genomic phenotyping. The latter is the DNA analysis of an unknown owner to identify physiognomic traits. These traits can be used for intelligence when a direct DNA match cannot be established. Developers in the public domain mainly focus on the hair, skin and eye color of unknown persons in order to narrow down groups of suspects to those with matching visible characteristics (Kjersem 2020). These profiles can also be combined with so-called biogeographic ancestry analyses, such as sub-Saharan Africa, East Asia, South Asia, Oceania, and the Americas. Some commercial developers go one step further: they attempt to use phenotypic data to create images of faces reminiscent of traditional police mugshots, which is currently a far more uncertain technique (see Schneider et al. 2019). A number of ‘cold cases’ have been solved after such images generated tips from the public (see, e.g., Canadian Press 2018). The company Parabon (2022), for example, argues that their software helped solve more than 200 cases. Such cases are invoked by commercial and some police actors to argue for their use despite the product’s methodological uncertainties. The rise of phenotyping in forensics has been addressed by several disciplines (cf. Toom et al. 2016; Schneider et al. 2019; M’charek et al. 2020; Hopman 2021; Engelmann 2022), but still needs criminological discussion.

In some ways, this comparison of Lombroso’s typing and phenotyping is problematic. The forensic sciences have professionalized procedures for validating measurements and visuals by means of hypothetical analysis and experiments. What is more, the project of forensics largely moved away from explaining criminality, to creating profiles for evidence and investigation. Still, the legacy of crime sciences in new profiling procedures cannot be denied. Both Lombroso’s typing and genetic phenotyping, we argue, reduce bodily complexity and function as markers in law enforcement and society at large. The paper’s argument, then, does not assess the scientific quality of the two phenotyping practices, but discusses the logics and meaning of typifying bodies in criminological domains.

In order to develop our argument, we begin with a brief introduction of the role of data archives and bodily visuals in criminology. In a second part, we describe the meaning of phenotyping in relation to Lombroso’s and genomic practices, as well as related data practices. This leads us to a discussion of biometric typing as a practice of marking and its consequences. We conclude with a summary of our argument, not least to emphasize the importance of continuing to reassess the role of visual types in the discipline of criminology and of exercising careful critique.

## Criminology and the Visual: of Analog and Digital Archives

Criminology has an intimate relationship to visualization (Brown and Carrabine 2021: 181). The work of scientists and police forces in the nineteenth century, specifically Césare Lombroso’s efforts of metricizing and visualizing bodies, come to mind. His ambitious project of organizing and archiving ‘visual markers of deviance’ created his controversial legacy not only as the ‘founding father’ of criminology, but also that of visual criminology (Rafter 2006). We contrast his archival work with a specific archive of the twenty-first century: forensic databases containing genomic profiles used for the creation of digital biometric images.

Both types of archives are not neutral storage spaces of visual data, but places where knowledge is ordered and generated by socio-technical practices for a variety of purposes (Thylstrup et. al. 2021). Derrida links the notion of the archive directly to the criminological context as the Greek *arkheion* is the place where documents of the law were stored, the place “from which *order* is given” (1998: 1, emphasis in original), a place of power. If archives and the visual representation of data in crime statistics, diagrams, maps and profiles are a constitutive part of (visual) criminology (Wheeldon 2021: 22), then we seek to turn visual criminology against itself. When visual approaches and their archives “simply reproduce existing power relations,” they need to be “challenged, transgressed, reversed, and reimagined” (Brown and Carrabine 2021: 171). Here, we emphasize that we need to bring analog archives into dialogue with digital archives. A critical engagement with practices of measuring bodies, of archiving, visualizing and communicating knowledge about crime throughout the years is then also a critical engagement with criminology’s history and present. As we shall see below, digital databases and big genome data enable a new type of inference about bodies that criminologists need to be able to analyze and discuss.

By looking at visualizations of bodies based on genomic information, we analyze the use of technologies that “render a picture of that which cannot be seen” (Brown and Carrabine 2019: 199) – at least not without digital mediation. This rendering expresses a desire for control and mastery at a new, molecular level (Rose 2000). *Visualizing the invisible* assumes here several meanings: it is not just a rendering of that which cannot be seen with the bare eye into an actual body image, but it is also an act of tying molecular and physiognomic traits to intangible, yet powerful notions of “latent social dangerousness” (Horn 2003: 59). Our article then follows Nicole Rafter’s call to consider *all* forms of making visual as relevant to criminology. This project should not only focus on the image, but also on practices, people and contexts of visualization (Young 2014).

When we direct our attention to forensic visualizations, the ethical question “What right have *I* to represent *you*?” (Levi Strauss 2003: 8, emphasis in original, cf. Carrabine 2012) involves a twofold reflexivity. The first one relates to the ethics of visual representations that co-constitute forensic gazes and understandings of crime. Since such forms of rendering visual are highly technologized and scientized, they easily evade ethical discussions. This was also the case for Lombroso’s visualizations at the time, where critical engagements addressed the choice of method (e.g., Rafter 2006) rather than the very concept of measuring bodies. In fact, Lombroso’s work was part of a much broader, international effort of scientizing crime (Becker and Wetzell 2006).

Asking, “What right do I have to represent you?” prompts a second moment of reflection, namely our own obligations as researchers when questioning these visual representations. What is our position as criminologists, researchers, authors when we join the project of critique? This question is particularly relevant considering that many forensic scientists are already aware of the limits of DNA analyses. So when we chose to ‘represent’ and critically discuss forensic techniques in this article, we acknowledged the high scientific standards of (most) forensic cultures. We also see that the forensic community cares to pay attention to potential pitfalls when communicating results to legal professions (Roux et al. 2022). We do, however, ask whether this critical awareness really moves beyond discussions on the choice of method and communication strategies. With our analysis, we point to those questions that scientific projects of visualization rarely address: What do these body images represent when they are tied to crime? Where and when do visualizations make powerful choices about identification that impact identity and society, and open doors for future socio-scientific practice? What is the ethos of such visuals?

## ‘Phenotyping’ and the Visualization of Suspect Bodies

Phenotyping is a specific form of visualizing the body. In fact, the original definition of the process dates back to Lombroso’s lifetime. A phenotype is the “sum total of the observable characteristics of an individual; type of organism distinguishable from others by observable features," (Harper, n.d.). The origin of the term is Wilhelm Johannsen’s “phaenotypus” (1909). He coined this term as a part of his heredity studies, where the phenotype results from the interaction of the genotype and environmental influences. It refers to ‘observable features’ that can be measured (orig. “messbare Realitäten,” ibid.: 123), including anything from physiognomy to the traits of a disease or, according to more recent discussions, behavior (Hofvander et al. 2009). Due to its emergence from heredity studies, phenotyping refers to *(epi)genetic and genomic* research. The etymology of the word, however, from Greek *phaínō* “to appear, show” and *túpos* “mark, type,” suggests that phenotyping includes much more. Digital phenotyping in epidemiology, for example, refers to the use of monitoring devices and big data in precision medicine for the creation of pathological knowledge (Engelmann 2022). Thinking about phenotyping more broadly, one may read Lombroso’s project of providing biological explanations for deviance as a form of phenotyping. His work is even discussed in relation to typing aggressive behavior (Sirgiovanni 2017).

Lombroso’s ‘phenotypic’ work was linked to the emergence of “statistics of illness and death” (Rose 2007: 4) which surfaced in the pathological anatomy of the early twentieth century. These gave rise to varied data such as medical images, maps, diagrams, and entire atlases, the visuals of which inspired typing and the quest for underlying formulas that could be found in the body. Lombroso’s ambition to create body types is emblematic of these trends and expresses the criminological project at the time.

Now that “phenotypically distinct conditions (…) appear to be related at the molecular level” (Rose 2007: 6), for example in the case of DNA analyses, the collection of data, the rendering of types and their relationship to crime, we argue, does not disappear, but is rearticulated. In his account of molecular biopolitics, Nikolas Rose traces how

(t)oday (…) biomedicine visualizes life differently. Life is understood and acted upon at the molecular level, in terms of the properties of coding sequences of nucleotide bases and their variations, the molecular mechanisms that regulate gene expression and transcription (ibid.: 5).

This molecular gaze, we suggest, produces its own forms of visualizing bodies that are both similar to, but also very different from Lombroso’s types. They involve more abstract, digital and microbiological data and visualizations, which nonetheless result in quite familiar depictions of bodily physiognomies.

Both Lombroso’s visual archives of ‘born criminals’ and today’s forensic DNA archives constitute endeavors in developing crime science further. These efforts of scientization profit from the appeal of the visual. Though underlying processes of measuring bodies are complex procedures, their visualization makes them accessible and renders them plausible (Rafter 2006). Lombroso’s use of media, for example, had its own pedagogy and explanatory power. His output was.

more visual and graphic than other theories of crime. Moreover, its photographs, maps, and drawings of measuring tools carried an assertion of scientific status, bolstering criminal anthropologists’ claims to objectivity. (Rafter 2006: 175-6)

Lombroso’s use of visuals was inventive. It was a new epistemological practice signaling the “idea of scientific prowess” (West 2017: 280) and creating a “new set of visual codes for criminality” at the same time (Rafter 2006: 177). In doing so, visualizations would not only produce knowledge about bodies, but they would endow bodies with a specific ontology (cf. Butler interviewed by Meijer and Prins 1998). Lombroso’s types endowed bodies with the ontology of anatomic causes for crime. Genomic phenotypes, as we shall see, endow bodies with the ontology of suspicion.

These ontologies were not based on images alone, but on a combination of different types of data. Indeed, Lombroso’s and today’s phenotyping efforts are also characterized by the rise of datasets, archives and forms of quantification that were and are new and typical for their relative era. Lombroso’s metric efforts, his collection of visual objects and his archives were unique in size and constantly expanded (Gibson and Rafter 2006a; Rafter 2014; West 2017).

Considered by Lombroso as “data” relevant to criminal identification (…) these objects included skulls, skeletons, pickled brains, photographs, wax effigies of “deviant” faces (Rafter 2009: 150)

One could consider Lombroso’s archive the ‘big data’ of his time. Today, phenotyping is based on ‘big genomic data’ (Murphy 2018) and digital archives—phenomena that could only emerge from contemporary technologies and storage capacities. Indeed, the role of devices and cutting-edge technologies (cf. West 2017) is intimately linked to the type of data and data visualization that can emerge. What photography and anatomic visuals were for Lombroso and others (Kemp and Wallace 2000), are next generation sequencers, biobanks and software today. Only the rendering of DNA into digital files and their storage in DNA databases could enable the transmission, comparison and large-scale correlation of genomic data for phenotyping (Kaufmann 2022). All of these new developments are and were in need of new expertise. While today’s research in molecular biology is not easily accessible to the general public, and at times not even to law enforcement (Roux et al. 2022), Lombroso’s medical and metric expertise was equally inaccessible to the general public at the time. The visual language of their outputs, however, mediates this inaccessibility.

It is crucial to remember the differences that characterize each practice. As mentioned before, Lombroso’s categorization and visualization efforts would not meet today’s scientific standards. Most important, the ambition of Lombroso’s work was to link visual body traits to crime, while today’s phenotyping is not (yet) aimed at establishing the body as a cause of crime. The two practices, however, produce bodily images based on biological traits. These visual types circulate in the domain of crime control where they perform similar politics. Hence, in order to discuss the epistemologies and ontologies of phenotyping, we will briefly introduce the two techniques.

### Lombroso’s ‘Phenotyping’

‘Lombrosian’ techniques are commonly understood as “the idea of identifying a criminal from his face” (Musumeci 2012: 132). Large parts of Cesare Lombroso’s studies concerned facial sizes, forms and textures, guided by the belief that morality is expressed through externally visible features. His project began, however, inside the body, at the skinless skull underneath the face. His earliest scientific endeavors drew on observation and measurements of cranial circumference, which was highly detailed, and noted in millimeters. The first edition of Criminal Man (1876) opens with cranial measurements of 66 skulls, which he categorized according to size:

[M]easurement of cranial circumference found very few criminal skulls that were particularly large (one of 580 mm [millimeters], two of 560 mm, one of 550 mm, two of 540 mm) or even normal in size (eight of 530 mm, thirteen of 520), but a high incidence of craniums that were microcefalic [*sic*] or abnormally small: thirty-nine out of sixty-five. More precisely, there were nineteen at 510 mm, twelve at 500 mm, and eight at 490 mm (Lombroso 1876 in Gibson and Rafter 2006: 45).

The purpose of the opening chapter was to show that the craniums of criminals were not ‘normal’, but different and could be told apart from those of the non-criminal. He used measuring instruments such as thermometers for temperature, dynanometers for force and weight, and broca stereographs for skull profiles, and statistical calculation to draw conclusions. These conclusions were not drawn on measurements alone: Lombroso had access to information about the lives of the people he was studying. Only when contextualized by social, behavioral information did his physiognomic observations create grounds for a positivist theory of crime. For example, in the analysis of Giuseppe Villella’s skull he combined the observation of a ‘small hollow at the base of the skull, and, underneath it, an enlarged segment of the spinal cord’ (Lombroso 1876 in Gibson and Rafter 2006: 139), with information about Villella’s alleged crimes of thievery and arson in order to assign Villella’s case to a criminal type. At the same time, his work was driven by the dictum that ‘anthropology needs numbers, not isolated, generic descriptions’ (Lombroso 1876 in Gibson and Rafter 2006: 53). By providing numbers on ‘criminal’ bodies, Lombroso connected appearance to behavior and the natural to the social sciences. Indeed, as described by Villa,

The series of anthropological anomalies (…) would then allow criminals to be classified by ‘type’. This would enable – theoretically and also preventively – identification of those due to organic predisposition, distinguishing them from the insane or from occasional criminals, or victims of passion or desire (2012: 16).

The baseline in Lombroso’s phenotyping efforts is three categories of human: ‘normal’, ‘criminal’, and ‘insane’. Lombroso never challenged the three types, but continued to develop new sub-categories within them. The separation between groups included not only visible features, but also other metric factors, such as strength, balance, or left-handedness, and ‘moral statistics’, such as “limited intelligence, weak memory, hallucinations, impulsivity, delusions of grandeur, irascibility, lying, theft, religious delusions, pederasty, perversity and masturbation,” which “so frequently leads to crime against persons” (Lombroso 1889 in Gibson and Rafter 2006: 251–2). In responding to criticism about the lack of statistical data, later editions of his *Criminal Man* included classification systems and descriptions of the experiments he had conducted. Such as a chart of armpit temperatures:

(…) Ottolenghi and I found that the armpit temperatures of fifteen criminals examined in our laboratory (at 3 PM) were:

- 97.88° 1 insane rapist
- 98.06° 2 epileptic thieves
- 98.96° 3 revolutionaries (1 was epileptic)
- 99.50° 6 who committed assault (1 was insane and 1 epileptic)
- 99.86° 2 murderers (1 was epileptic)
- 101.84° 1 swindler

The average temperature was 99.32°. Overall, these temperatures were slightly above normal. (Lombroso 1889 in Gibson and Rafter 2006: 237)

In a gradual move from the physiological to the psychological, Lombroso expanded his ‘criminal type’ from types of crimes to include the broader social categories, such as sub-categories of *madness* (e.g., moral epilepsy, which was considered a crime). He either examined people directly or looked at photographs of people whom he knew had expressed signs of madness and described their “degenerative characteristics,” such as "jug ears, enlarged sinuses, large jaws and cheekbones, sullen or crossed eyes, and thin upper lips” (Lombroso 1889 in Gibson and Rafter 2006: 272). He then concluded that those were the traits of madness. This created a system where new people could be added to already existing categories. People who shared similar visible traits were presumed to share their mentality and dangerousness. Still, he was criticized for “bending his data to fit preconceived theories” (Gibson 2006: 139) and being selective in choosing which available data would lay the grounds for a particular theory (Gibson and Rafter 2006: 395). His contemporaries, students and self-proclaimed ‘disciples’ said that “he was a genius at guessing, but when he had to expound, to demonstrate, he was lost” (Onoranze 1921: 729 in Villa 2012: 11).

Lombroso’s descriptions of physiognomic traits echoed the scientific and Darwinistic discourse at the time. For instance, Lombroso described some skulls as “monkeylike” (Lombroso 1876 in Gibson and Rafter 2006: 45). From this, he gathered that the intelligence and behavior of the person would resemble that of “prehistoric” people (ibid.: 49).

Lombroso’s ‘phenotyping’ project always began at what was visual—sizes, shapes, colors, weight—and moved inwards and outwards, between the psychological and the physiognomic, in continual exchange. Through codifying and systematizing data and categorizing the people he was looking at, he arrived at a comprehensive biometric visualization of risk and deviance.

### Genetic Phenotyping

Today, different biometric data have become a base for categorization: genomic information. Phenotyping is used to analyze those genomic regions that are expressed as physiognomic features. Typing, here, is a biochemical as well as a digital process. This paper focuses on the digital process, that is the correlation and categorization of genomic data for predictions.

Basic solutions for phenotyping developed by non-commercial forensic actors mainly focus on the prediction of eye-, hair- and skin color (cf. ISFG, n.d.), and sometimes age predictions. Commercial developers, on the other hand, promise more complex results, which is reflected in the marketization of their products. The company *Parabon* offers a tool called “Snapshot” that provides police officers with an image, much like an identikit picture derived from DNA (cf. Parabon: n.d.). The idea is to use such information when there is no DNA match available. Genomic predictions, so the idea goes, would then allow for the investigation of individuals with a typical physical appearance. Here, Parabon’s “Snapshots” could be made publicly available to generate tips. This, it is claimed, has led to arrests in several high-profile criminal investigations (Associated Press 2017; Canadian Press 2018; KPLC News 2017). The difference to Lombroso’s ambition is that types are not produced to develop *profiles of deviance*, but to produce profiles that, when tied to DNA from a crime site, are meant to lead the police to *potential suspects*. The fact that such images already play a role in investigations today underlines the importance of discussing these trends in criminology.

In order to create a phenotype, the coding regions of the genome need to be sequenced, which is to identify the genome’s A’s, C’s, T’s, and G’s, i.e., their bases. Sequencing these regions is relevant because they encode proteins for different bodily functions. Alterations in the code are called Single Nucleotide Polymorphisms (SNPs) which—in short—are genetic markers for a specific bodily trait. In order to know which marker is linked to a specific physiognomic trait, one would need a large database of individuals whose pheno- and genotype is known. From this information, *inferences* are made as to which SNP correlates with what phenotypical trait. For the sake of creating a visual profile, continuous readings of eye-, hair-, and skin color are categorized in accordance with *type*, e.g., ‘white’, ‘brown’ or ‘black’ skin. Such gradual differences, needed for predictions, are created manually: someone has to decide at what shade the category of ‘white’ skin or ‘blue eyes’ begins and ends. Such decisions are fed back into the software. How casually categories are created, sometimes by non-experts or volunteers, are described by Hopman (2021), who also critiques such acts of categorization as a simplification of phenotypic variation. Indeed,

(t)he decision to articulate differences in skin variation through the categories of white, intermediate and black is a decision that valorises everyday racial categories over more nuanced possibilities (2021: 8).

There are ongoing efforts to use genetic phenotyping for making predictions about the morphology of the faces of unknown suspects, too. This practice is, however, more contested (for a discussion, see Wienroth 2020). Morphological predictions are needed, for example, to create facial identikit images, as is the case with “Parabon Snapshot.” Here, a similar reference database is built with individuals whose morphologies are captured biometrically and whose genotype is known. In order to organize the data about morphologies, templates of ‘typical’ faces (e.g., a ‘typical’ Northern European face) are developed. For the sake of standardization, “irregularities” in the reference faces are removed (see Hopman 2021: 10–12). An algorithm then correlates the (human-made) morphological categories to SNPs. The result is a *derivative* (Amoore 2011). It is a statement about physiognomic expression that has genomic data at its base, but is no longer interested in the specificities of it (ibid.). This is emphasized by the very fact that specificities are cleaned away in order to predict better ‘types’. While the inferential agency of the algorithm is crucial in the making of phenotypic predictions, human interaction and decision-making is necessary for developing metric parameters; they are crucial to the typing process. These particular moments of developing metric categories are similar to the work of Lombroso when he decided at what size a ‘normal’ skull circumference began and ended.

There are also ongoing efforts to locate non-pigmentation markers in DNA, like baldness (Liu et al. 2016), age (Schneider et. al. 2019) and voice (Lippert et al. 2017). These efforts illustrate that the project of scientizing police work and integrating it with biometry is—and will be—continued. Creating types, and conveying knowledge about types via visuals, is central to this project. Here, Lombroso’s and genomic phenotyping emphasizes how scientific techniques and technologies are always dependent on human decisions about visual categories. However, few of such decisions are visible to lay people. We will now turn to the further implications of categorizing and visualizing types, focusing on their social life. After all, any such typologies are supposed to be used in law enforcement, meaning socio-political practices.

## ‘Phenotyping’ as a Practice of Marking

Visuals, as argued above, are part of how crime is imagined and framed within society. Phil Carney (2015) offers here a term that allows us to discuss this political nature of visualization in the context of criminology. From Foucault’s body of work on the punitive society, he develops an argument on visualization as a practice of *marking*. To mark is to inscribe power on the body as a form of punishment. He discusses, amongst many examples, the early rise of photography to record the faces of convicts as a punitive form of visual marking (Carney 2010, 2015). This use of photography is compared to contemporary developments:

Most recently, the photographic medium has revived an almost Lombrosian use of the photographic image, creating stigmatized categories of the underclass in a punitive spectacle of ‘meth’ (methamphetamine) users. (Carney 2015: 242; Linneman and Wall 2013).

We appropriate this concept of marking here not as a form of punishment, but as a practice of (re)presenting the criminal or suspect body. Any phenotype, we argue, is also a visual marking of the body: tying bodies to the context of criminality is (bio)political as it is also a form of reducing the body for the sake of governance. Renzo Villa describes Lombroso’s work as a reductionist program (2013), and Nikolas Rose argues that the early twentieth century was focused on physical signs of abnormality and “visible *marks* of criminality” (2000: 8, emphasis added). It was a technique of presenting the criminal being to the public and as a subject of law enforcement.

Today, we have moved from twentieth century *anatomic markers* that were meant to reveal *causes* of criminality to metaphorical and literal *genetic markers*. These are used to identify *suspects* and their physiognomic type, representing their—albeit temporary—legal status. As mentioned above, acts of rendering visual and of marking the body in the context of law enforcement are constitutive and productive of bodies. Already early visualizations endowed bodies with ontology: “Through their discursive and visual texts, criminal anthropologists created a new being: the born criminal” (Rafter 2006: 177). DNA, too, has a strong ontological image: it is considered a gold standard in forensics. Popular narratives about DNA present it as the ultimate locus of individuality (*It’s in my DNA to [you name it]!*). Ian Hacking’s ‘genetic imperative’ – “the drive to find genetic markers in humans” (2006: 99), receives a new reading here. He writes that “people can hardly avoid thinking of their genetic inheritance as part of what constitutes them, as part of who they are, as their essence” (Hacking 2006: 92). Daniel Navon mirrors the above points about identity when he speaks about the genomic production of “new kinds of people” (Navon 2011: 220). He refers to Ian Hacking’s (1995; 2007) ‘looping of human kinds': “how the ways in which experts categorize and treat people interacts with the way people are to mutually transform one another” (Navon 2011: 219).

Both Lombroso’s and genomic phenotyping are practices of marking, but differ in terms of the marker and what exactly their mark represents. That is to say, the legal status ascribed to the respective ‘type’ differs. Lombroso’s phenotypes mark the nature of the criminal being. This mark emerged from a *biology of depth*. The epistemological power of Lombroso’s visuals was drawing on the medical authority of anatomy. They stand for a “deep ontology” that set out to “discover the underlying laws that determined the functioning of closed living systems” (Rose 2007: 7). Genomic phenotypes are physiognomic markers for suspicion. They denote a “flat ontology” that is concerned with maps and circulations (ibid.) and derivative logics (Amoore 2011). Today’s visuals draw on the scientific image of molecular biology and computation, a key feature of which is forward vision. As we will see below, genetic phenotyping is a prediction, which is (for now) used for intelligence rather than uncovering causalities.

### The Logics and Effects of Marking

As mentioned earlier, we do not forward a critique of the scientific quality or validity of phenotyping. We discuss the role of visuals, of ‘phaino’, and the creation of types in the context of crime governance and the related power of marking bodies. Here, Lombroso and modern phenotyping produce different types of bodily markers. Lombroso “employed images on the premise of realism” (West 2017: 280), which expresses his belief in the epistemological power of the visual. This work with visuals was corroborated by a range of other epistemic objects and data practices, such as the creation of an archive of images and anatomic relics of convicts, or his techniques of measuring and the equipment to do so. With these as a base, we (re-)emphasize that Lombroso set out to identify “visible *marks* of criminality” (Rose 2000: 8, emphasis added), creating markers of *deep ontology*. And he translated these marks directly to the *social* context of *marking*. Based on his data, he concluded that

there is nearly always something strange about their appearance. It can even be said that each type of crime is committed by men with particular physiognomic characteristics [and] this may explain why the overall appearance is neither delicate nor pleasant. (Lombroso 1876 in Gibson and Rafter 2006: 49).

The marker of the ‘born criminal’ emerged from Lombroso’s anthropological and biological project, disciplines that Paul Gilroy criticizes as co-responsible in rendering discriminatory categories “epistemologically correct” (Gilroy 2000: 58).

How does Lombroso’s marker of the ‘born criminal’ relate to the marker that genomic phenotyping produces today? Some defend genomic practices in the context of law enforcement and prediction as non-deterministic (cf. Sirgiovanni 2017). In his critique of genomic practices, Nikolas Rose argues that as opposed to twentieth century eugenics, today’s phenotyping is not about ‘biological reductionism’. A new subjectification occurs instead, bearing a collective understanding of the morally responsible individual. This entails that risky individuals are managed and controlled via prevention programs (2007).

On the one hand, one could argue that the marker of suspicion produced by genomic phenotyping indeed follows the *flat ontology* that Rose describes: instead of linking the genome to causes of crime, genomic phenotyping engenders a form of subjectfication where correlation leads to the temporary status of suspicion. In that respect Rose’s observations hold true for forensic phenotyping and its impact today.

We do want to argue, however, that the logic of types and especially their *visualization as specific bodily characteristics* still function as a reduction. And this reduction produces additional effects in their role as a social marker. Not only are bodily complexities reduced to racial categories, as argued by Hopman (2021), but such reductions, again, enable expansive policies of marking. Phenotyping based on eye-, hair- and skin-color marks entire groups, as illustrated with the example in the opening of this article. It favors the investigation of a body type. In practice, this form of marking easily lends itself to ascribing a more inherent notion of suspicion to a specific body type, especially when bodies have already been subject to a history of discrimination and racism in police work (Benjamin 2019; Abu El-Haj 2007). Again, the visualization of body types is especially decisive here. Indeed, Victor Toom et al. emphasize the role of externally visible characteristics (EVC) here:

EVCs can easily render minority groups into suspect populations since the predominant group living in a particular area or country is often too large to investigate (…). This effect of operational use must be further contextualized by limitations to effective and objective use of information by law enforcement agencies and individuals and the likely role of cultural bias, for example in policing practices and media stereotyping of the genetic suspect (2016; cf. Duster 2004).

Paul Gilroy’s work comes to mind, who argues that ‘race’ is always based on more than linguistic conceptions, but also depends on visual and optical imaginaries (2004). The power with which such optics function as markers, we argue, changes when integrated with the scientific imaginaries of molecular biosciences. It makes a difference that the police investigation of types is no longer linked to classic identikit pictures only, but to *genetic* markers with a strong ontological and scientific image. Such markers influence police practice (cf. KPLC news, 2017; Associated Press, 2017, Canadian Press, 2018) and express the epistemic status of investigating specific groups. The logic of ‘types’ essentializes bodies. What is more, genetics and the ‘types’ they generate are linked to ambiguous and highly sensitive contexts when used in the domain of crime and crime governance. The effects of this use are concrete: in investigations genetic profiles reinscribe race into police practice and render entire groups suspect (M’charek et al. 2020). Nikolas Rose (2000) and Nadia Abu El-Haj (2007) also show how genetic predictions lead to differential treatment of people (e.g., prevention programs), where genetic profiles move between different societal domains.

We also point out that some developments in phenotyping in fact open the door for a combination with deep ontologies, for reviving the *causal* relation between crime and physiognomy by means of *correlation*. Scientific ambitions to progress beyond eye-, hair- and skin color have already been mentioned. In tandem with technological development and new areas for application, some research projects in genetics and psychology focus on neurochemical profiles with the aim of correlating them with deviant, ‘antisocial’ behavior and aggression (e.g., Dermott et al. 2009; Wertz et al. 2018; Hill et al. 2021). The analysis of coding regions for ‘typing’ is a part of such predictions. When tied to biosocial theories of explaining crime, such phenotyping efforts pave the way for practices of marking that have to be assessed critically vis-a-vis trends in the distant and recent past.

## Conclusion

Crime and control, as Eamonn Carrabine (2012) argues, cannot be divorced from their visual representation. Hence, we emphasize that criminology and its related sciences need to conduct self-assessments and critically evaluate the relations between crime, control and visual representations. The visualization of bodies in the context of law enforcement is a particularly sensitive issue. In that spirit, we turned to the visual representation of *body types* in these domains. This is a relevant phenomenon in the history–but also the present–of criminology, where issues of marking keep resurfacing in new gowns.

The analysis of body types and their marking are never isolated activities. They are embedded in specific historical and socio-technical environments and driven by professional ethics, aims and commercial markets. Making statements about bodies is a collaborative practice of scientists, machines, forensic and police professions, all of which draw on the authority of the visual, of (big) datasets, of state-of-the-art technology, and of scientific technique. Keeping this complexity in mind, we studied and compared two emblematic forms of visualizing bodies in the tradition of scientizing criminology: Cesare Lombroso’s ‘phenotyping’ and genomic phenotyping.

Both of them are biometric practices interlinked with human decision-making processes. Since phenotyping conveys criminological knowledge in ‘accessible’ visualizations, they are also powerful techniques of marking: specific body types receive the marker of being criminal or the marker of suspicion. Marking, one could argue, is even the *intention* of both techniques. Lombroso’s markers are ontologically “deep” (Rose 2007) in the sense that body types are linked to underlying causes of criminality. His typology of ‘born criminals’ expresses this logic. Today’s phenotypes are “flat” (ibid.) as they embody the logic of molecular circulations and data correlations, the aim of which is not to analyze causes, but provide actionable knowledge to the police. Phenotyping marks a specific physiognomy as suspicious, at least for the time of investigation.

Though their underlying scientific effort differs, both markers have something in common. They reduce human complexities, which impacts crime control and society at large. Several aspects play into the performativity of markers: they are tied to the scientific status of typing, the types are visual in nature, thus presenting knowledge in a simplified fashion which directly relates to their purpose of navigating crime control. However, the human decisions and the values enacted by these decisions are not (always) represented in such visuals. The *scientific image* (in both senses of the word) especially contributes to this relative invisibility of values.

Lombroso’s systematization of ‘degenerative characteristics’ is a prime example of using datasets and scientific means to render discriminatory categories “epistemologically correct” (cf. Gilroy 2000). Visualizing and categorizing the ‘abnormal’ body as ‘criminal’ emerges here as a double marker. The types mark bodies as deviant: they are different from the rest of society in terms of their physiognomy *and* their social behavior.

The ‘abnormal’ body in genomic phenotyping is more covert and complex. It is enacted in the making of the database, where non-standard faces are cleaned (Hopman 2021), which is done to translate them into the *normal, average* body images needed for predictions. The performative power of the genomic marker lies not in the production of the criminal, but of the potential suspect. While these markers could be considered less essentializing than Lombroso’s, genomic phenotyping is a practice that combines biology associated with individuality, personality and identity with crime control. DNA, we argue, emerges as the ultimate marker. Not only the body’s anatomy, but its molecular basis is securitized. Using an individual’s genetic makeup to predict their ‘type’, visualize it and place it in the context of law enforcement is also a double marker, but in a different sense: in marking bodies as ‘normal’, genomic phenotyping brings individuals *and* entire groups into the realm of suspicion.

Our analysis is not only a critical evaluation of the role of visualizations in specific practices of crime control. It goes back to the emergence of criminology as a discipline and the role bodies play here. This relationship between crime control and the body will prevail and take ever-new forms. This requires concrete engagement: visual technologies need to be assessed in terms of their simplifications, the balance of actionable knowledge and broader societal implications, as well as their scientific histories. This assessment is not limited to *criminological* analyses of socio-scientific technologies. *Engineers* and *proponents of genetic profiling*, too, have social and ethical responsibilities when developing solutions—especially when such technologies are intended for crime governance. Some representatives of the forensic community have already begun to address these issues (Roux et al., 2022), but the ethos of phenotyping warrants continued, profound assessments and interdisciplinary dialogue.
